# Structure and Function of Centromeric and Pericentromeric Heterochromatin in *Arabidopsis thaliana*

**DOI:** 10.3389/fpls.2015.01049

**Published:** 2015-11-30

**Authors:** Lauriane Simon, Maxime Voisin, Christophe Tatout, Aline V. Probst

**Affiliations:** CNRS UMR6293, INSERM U1103, Clermont University, GReD, Aubière, France

**Keywords:** centromere, chromocenter, histone variants, 3D nucleus, lamina, nuclear envelope

## Abstract

The centromere is a specific chromosomal region where the kinetochore assembles to ensure the faithful segregation of sister chromatids during mitosis and meiosis. Centromeres are defined by a local enrichment of the specific histone variant CenH3 mostly at repetitive satellite sequences. A larger pericentromeric region containing repetitive sequences and transposable elements surrounds the centromere that adopts a particular chromatin state characterized by specific histone variants and post-translational modifications and forms a transcriptionally repressive chromosomal environment. In the model organism *Arabidopsis thaliana* centromeric and pericentromeric domains form conspicuous heterochromatin clusters called chromocenters in interphase. Here we discuss, using *Arabidopsis* as example, recent insight into mechanisms involved in maintenance and establishment of centromeric and pericentromeric chromatin signatures as well as in chromocenter formation.

Centromeres are essential chromosomal structures that were first defined as central restrictions of the mitotic chromosomes that function in chromosome segregation during cell division. Except for *Saccharomyces cerevisiae*, centromeres are not defined genetically by a specific DNA sequence but rather epigenetically by a particular chromatin environment and the presence of the specific histone variant CenH3. The centromeric and the surrounding pericentromeric chromosomal regions form heterochromatin domains that remain condensed during interphase (Heitz, 1928) and in some species like *Arabidopsis thaliana* these are clustered into chromocenter structures (Figure 1; Fransz et al., 2002). Here we discuss our current knowledge concerning sequence composition, chromatin features and interphase higher-order organization of centromeric and pericentromeric regions into chromocenters, referring to the centromeric region specifically as the part of the chromosome involved in kinetochore formation, while we refer to the pericentromeric domains as the adjacent chromatin regions (according to Gent and Dawe, 2011).

**FIGURE 1:**
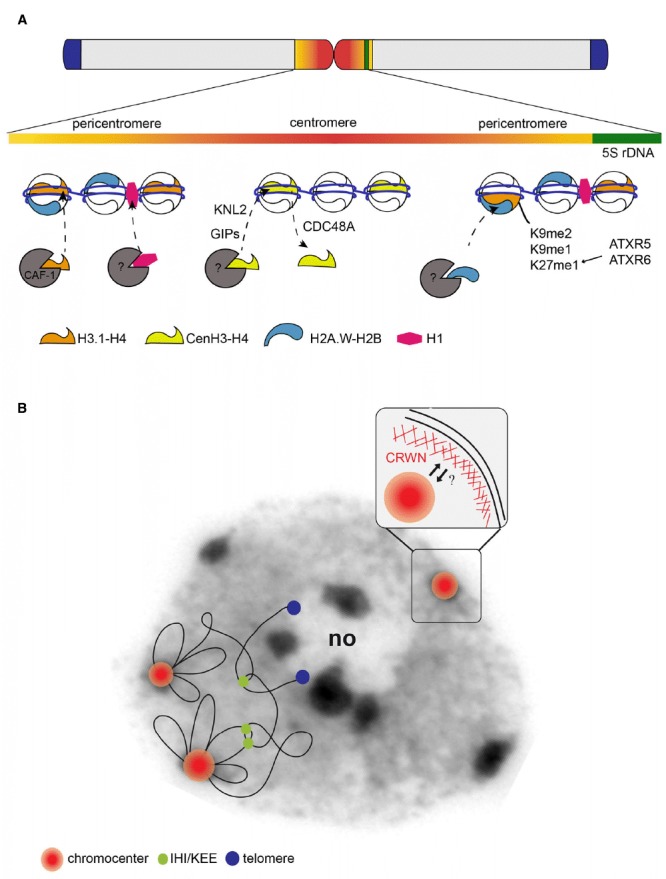
**(A)** Model of an *Arabidopsis thaliana* chromosome. Enlargement of the central part of a chromosome (top) shows the sequence composition of the centromeric (red, mainly consisting of repetitive 180 bp repeats) and the pericentromeric region (orange, containing interspersed repeats, transposable elements and their derivatives) embedding a 5S rRNA gene locus (green) as well as their chromatin composition. The pericentromeric domains are enriched in the canonical histone H3.1 (orange), the H2A.W variant (blue) and the linker histone H1 (pink) as well as in repressive histone modifications such as H3K9me1/me2 and H3K27me1. The centromere is defined by enrichment in the H3 variant CenH3 (yellow). Whether CenH3 nucleosomes form large blocks or are interspersed with nucleosomes composed of canonical or specific variant histones and which histones are incorporated as placeholders during replication remains to be determined. CenH3, H1 and H2A.W are deposited by yet unknown histone chaperones. GIP1/GIP2 and KNL2 play a role in CenH3 deposition. In specific cell types, CenH3 is actively removed by the AAA-ATPase CDC48A. **(B)** Model of the organization of a chromosome in nuclear space overlaid on a DAPI stained image of an *Arabidopsis* leaf nucleus. Centromeric and pericentromeric regions of the 5 *Arabidopsis* chromosomes are tightly packed into chromocenters (red/orange). Chromocenters structure the chromosome in nuclear space by anchoring proximal euchromatic loops, while distal chromosomal regions tend to cluster with telomeres (blue) next to the nucleolus (no). Interactive heterochromatic island (IHI)/KNOT engaged element (KEE) regions, identified in Hi-C maps, form additional intra- and inter-chromosomal contacts (green). Enlargement at the top right shows the nuclear envelope coated internally by a lamina-like structure, which includes CRWN proteins. Chromocenters tend to localize at the nuclear periphery, but the physical link between nuclear envelope components or the nuclear lamina-like components remains to be identified.

## Sequence Composition of *Arabidopsis* Centromeric and Pericentromeric Regions

Centromeric sequences consist in most organisms of short repetitive DNA sequences arranged in tandem and/or transposable elements (Plohl et al., 2014). New centromeres can emerge from anonymous sequences but they gradually incorporate repetitive arrays (Han et al., 2009; Plohl et al., 2014) suggesting that DNA repeats may be a preferred DNA environment for centromere formation. In *A. thaliana*, centromeric regions mainly consist of a 178 bp long sequence also called AtCon, pAL1, or 180 bp (Kumekawa et al., 2000, 2001; Nagaki et al., 2003) that is arranged in head-to-tail tandem repeats ranging from ∼0.4 to 3 Mb (Figure 1A). The 180 bp repeats are interrupted by a 398 bp fragment of the Athila2 LTR called 106B (Thompson et al., 1996). While highly similar sequences are found on all chromosomes, some 180 bp repeat variants are specific to one centromere (Heslop-Harrison et al., 1999). Centromeres are flanked by a pericentromeric region, which in *Arabidopsis* contains various types of repeat sequences such as Athila retrotransposons, 500 bp and 160 bp repeats (Bauwens et al., 1991), “Transcriptional Silent Information” (TSI) composed of the 3′ half of an Athila retrotransposon (Steimer et al., 2000) and on some chromosomes the 5S rDNA clusters (Fransz et al., 1998; Cloix et al., 2000). Most repetitive sequences from the pericentromeric region and transposons are kept silent, while others like the 5S rRNA gene clusters (Cloix et al., 2002) are highly transcribed. Despite the efforts in genome assembly (Schneeberger et al., 2011), the establishment of the exact reference sequence for these highly repetitive chromosomal regions remains a challenge for the future.

## The Central Role of CenH3 in Centromere Definition

A high frequency of DNA repeats is not sufficient to define centromeres (Han et al., 2006; Birchler et al., 2010); instead, centromeres are determined by a specific chromatin environment. The basic subunit of chromatin, the nucleosome, comprises 146 bp of DNA that wrap around an octamer of core histones H3, H4, H2A, and H2B. A specific histone variant, called CenH3, replaces the canonical H3.1 in centromeric nucleosomes. CenH3 is enriched at 180 bp repeats as shown by Chromatin Immunoprecipitation (ChIP) and Fluorescence *in situ* hybridization (FISH) experiments (Nagaki et al., 2003; Shibata and Murata, 2004). In agreement with its central role in centromere definition, homozygous *cenH3* mutants are lethal and plants expressing RNAi constructs leading to reduced CenH3 levels show meiosis defects, partial sterility, and in older plants an increased 4C:2C ratio indicating G2 arrest (Lermontova et al., 2011). CenH3 proteins evolve rapidly, as example 23 out of 178 amino acids differ between the closely related species *A. thaliana* and *A. arenosa* (Talbert et al., 2002). The N-terminal tail is substantially longer compared to the canonical H3.1 or the variant H3.3 and particularly divergent between species, revealing adaptive evolution with the species-specific centromeric repeats (Talbert et al., 2002; Maheshwari et al., 2015). An additional domain involved in adaptive evolution is the histone fold domain including the loop 1 region that makes multiple contacts with DNA (Cooper and Henikoff, 2004). This histone fold domain has been found sufficient for CenH3 loading at centromeric sequences (Lermontova et al., 2006) and a single point mutation close to the loop1 region reduces CenH3 loading substantially (Karimi-Ashtiyani et al., 2015). While plants carrying this loading deficient CenH3 are fertile when selfed, backcrossing to wild type (WT) plants leads to haploid and aneuploid progeny, retaining only the WT CenH3. Unexpectedly, even CenH3 from distant monocotyledon species can complement *A. thaliana cenh3* mutants (Maheshwari et al., 2015). These results differ from other studies using N-or C-terminal GFP tagged versions of heterologous CenH3, where only tagged CenH3 from a closely related species was properly targeted or functionally complemented a *cenH3* mutant (Ravi et al., 2010). This shows that a GFP tagged CenH3 version is not functionally equivalent, as the large GFP tag may interfere with proper CenH3 loading or the assembly of kinetochore proteins.

Together, these studies underline a central role for CenH3 in centromere definition. Fast co-evolution of CenH3 and centromeric repeats is proposed to contribute to reproductive isolation and speciation (Ma et al., 2007; Plohl et al., 2014). Understanding these key mechanisms may have major application in breeding programs when interspecific crosses between cultivated species and their WT relatives are involved.

## CenH3 Deposition

CenH3 needs to be deposited in a controlled manner to avoid mislocalization to ectopic sites (Lacoste et al., 2014) that might seed neo-centromeres (Shang et al., 2013). Appropriate incorporation of CenH3 is therefore controlled by specific histone chaperones. In mammals, CenH3 is deposited post-mitotically (Jansen et al., 2007), while CenH3 nucleosome assembly takes place in G2 phase in *Arabidopsis* (Lermontova et al., 2006). In contrast to mitotic nuclei, meiosis includes also a post-divisional loading step during interkinesis (Schubert et al., 2014) and is associated with a specific loading pathway or quality check that eliminates modified CenH3 proteins (Lermontova et al., 2011; Ravi et al., 2011). No functional homolog of CenH3 chaperones known in humans (HJURP, Dunleavy et al., 2009; Foltz et al., 2009), Drosophila (CAL1, Chen et al., 2014) or yeast (SCM3, Camahort et al., 2007) has yet been identified in plants. In contrast, a homolog of yeast Mis18, which is implicated in forming the correct epigenetic context for CenH3 loading (Hayashi et al., 2004), has been identified in *Arabidopsis* and termed KINETOCHORE NULL2 (KNL2; Lermontova et al., 2013). KNL2 is mainly expressed in meristem tissues similar to CenH3 and except during mitosis localizes to centromeres during the whole cell cycle including G2 phase when CenH3 is loaded. Loss of KNL2 negatively impacts CenH3 expression and deposition (Lermontova et al., 2013), but also reduces DNA methylation and affects histone methyltransferase expression, suggesting that the chromatin context of centromeric or pericentromeric sequences repeats may play a role in CenH3 loading. Furthermore, recent work suggests a role for the γ-tubulin complex protein 3-interacting proteins (GIPs) in CenH3 loading or maintenance at centromeres (Batzenschlager et al., 2015). GIP proteins are found in a complex with CenH3 and a double *gip1 gip2* mutant shows reduced intensity of CenH3 signals, centromere cohesion defects and aneuploidy, despite increased levels of KNL2 in the *gip1 gip2* mutant background (Batzenschlager et al., 2015). Given that the CenH3 deposition machinery evolved rapidly and involves distinct players in yeast, Drosophila, mammals and plants, it can be speculated that GIP proteins are part of a plant-specific pathway contributing to CenH3 assembly.

Interestingly, CenH3 is associated only with specific subsets of 180 bp repeats (Shibata and Murata, 2004) and these are hypomethylated (Zhang et al., 2008) compared to other subsets of 180 bp repeats that are hypermethylated, enriched in H3K9me2 and associated with the canonical histone H3.1 (Stroud et al., 2012; Wollmann et al., 2012; Vaquero-Sedas and Vega-Palas, 2013). To which extent CenH3 containing nucleosomes are interspersed with nucleosomes containing canonical H3.1 or its variant H3.3, and which of the CenH3 types is deposited as placeholder upon chromatin assembly during S-phase, remains to be elucidated in plants. Furthermore, the identification of the histone chaperone involved in CenH3 deposition and further characterization of the role of GIP proteins will be critical to better understand how CenH3 is specifically targeted to centromeric repeats.

While controlled CenH3 deposition is important, in some differentiated cells, CenH3 is also actively removed, such as in the vegetative pollen nucleus that does not contain visible CenH3 enrichment at centromeres compared to the sperm cell nuclei (Ingouff et al., 2009; Schoft et al., 2009). In the vegetative nucleus, CenH3 is sumoylated and removed by the AAA-ATPase molecular chaperone CDC48A to be targeted for proteolysis (Mérai et al., 2014).

## The Pericentromeric Region

The CenH3 containing centromere domain is flanked by pericentromeric heterochromatin that is highly DNA methylated, shows more regular nucleosome spacing than euchromatin (Chodavarapu et al., 2010) and is characterized by inaccessibility to DNAse I (Shu et al., 2012). Pericentromeric nucleosomes carry histone modifications repressive for transcription such as H4K20me1, H3K9me1, H3K9me2, and H3K27me1 (Tariq et al., 2003; Naumann et al., 2005; Fransz et al., 2006; Roudier et al., 2011; Shu et al., 2012; Sequeira-Mendes et al., 2014). As an example, H3K27me1 loss is associated with release of transcriptional silencing of TSI and certain transposons in the pericentromeric region (Jacob and Feng, 2009) as well as over-replication of pericentromeric sequences (Jacob et al., 2010). The histone-methyltransferases ATXR5 and ATXR6 preferentially mono-methylate lysine 27 of the canonical histone H3.1 (Jacob et al., 2014), which is highly enriched in pericentromeric regions (Vaquero-Sedas and Vega-Palas, 2013). Furthermore, plants deficient in the Chromatin Assembly Factor 1 (CAF-1) complex that deposits histone H3.1 in a replication-coupled manner in mammals (Smith and Stillman, 1989; Tagami et al., 2004) show stochastic reactivation of TSI and CACTA transposable elements (Takeda et al., 2004; Ono et al., 2006). Additional core histones also exist as specialized variants enriched in heterochromatin such as the H2A.W variants H2A.W.6, H2A.W.7 and H2A.W.12 (Yelagandula et al., 2014), which colocalize as RFP fusion proteins with H3K9me2 at pericentromeric regions by microscopy. Simultaneous loss of H3K9me2 enhances the phenotype of double h2a.w.6 h2a.w.7 mutants and leads to increased expression of certain transposons suggesting that histone/DNA methylation and H2A.W incorporation present two parallel pathways involved in heterochromatin maintenance. Therefore, an important role can be assigned to the incorporation of specific histone types in the establishment of the particular chromatin environment of the pericentromeric region. Canonical or histone variants affect chromatin organization both through their inherent physico-chemical properties and through their specific post-translational modifications that might be set in a nucleosomal context, e.g., H3K27me1 by ATXR5 and ATXR6 (Jacob et al., 2014), or already during assembly (Loyola et al., 2009) and synthesis (Rivera et al., 2015) of the respective histone as recently described in mammals. Despite the advances in the description of the pericentromeric heterochromatin signature, not much is known whether and how pericentromeric heterochromatin contributes to centromere function in plants. Chromatin or sequence features of the pericentromeric domain may play a role in loading of CenH3 at the centromere as it is the case in fission yeast (Folco et al., 2008; Catania et al., 2015) but this remains to be investigated.

## Organization of Centromeric and Pericentromeric Chromatin into Chromocenters

In interphase nuclei, FISH experiments indicated that centromeric and pericentromeric repeats cluster together in chromocenter structures (Fransz et al., 2002). Recent Hi-C analyses confirmed that repeated sequences are grouped together and revealed further intra and inter-chromosomal interactions, Figure 1B. Multiple reasons have been brought forward to explain the particular organization of centromeric and pericentromeric sequences into chromocenters: the clustering may compartmentalize silent chromatin away from euchromatin, help concentrate chromatin modifiers setting repressive chromatin marks or coordinate replication of this domain in time and space (Heitz, 1928; Quivy et al., 2004; Almouzni and Probst, 2011). Chromocenters are not randomly organized in nuclear space but instead preferentially localize into the most outer zone next to the nuclear periphery (Fransz et al., 2002; Fang and Spector, 2005; Andrey et al., 2010; Poulet et al., 2015). To date, there is no clear explanation for this preferential localization and several hypotheses can be proposed. First, this organization can be the result of non-specific forces acting on heterochromatin because of its elevated thickness and rigidity in respect to euchromatin (Cook and Marenduzzo, 2009; de Nooijer et al., 2009). Second, peripheral position may be advantageous to allow rapid contact between the centromere and microtubules at the beginning of cell division. Interestingly, GIP proteins have been shown to localize to both sides of the nuclear envelope and close to the chromocenters and may therefore be seen as good candidates to connect the microtubule machinery and the centromeres upon nuclear envelope breakdown (Batzenschlager et al., 2014). The identification of the structural components linking heterochromatin to the nuclear periphery is an active area of research in plants and the lamin-like structures including CRWN1-4 (CRoWded Nuclei) proteins are intriguing candidates (Dittmer et al., 2007; Fiserova et al., 2009; Goto et al., 2014). Indeed, *crwn1 crwn2* mutants show reduced nuclear volume and increased chromocenter clustering, while chromocenters are more dispersed in *crwn4* (Dittmer et al., 2007; Wang et al., 2013a; Poulet et al., 2015). Hi-C data in *crwn1* and *crwn4* mutants reveal higher chromosomal compaction and increased interactions among pericentromeric regions, which reflects the altered chromocenter organization detected by FISH (Grob et al., 2014). Altered chromocenter organization was also observed in *syn4* and *cap-d3* mutants, lacking subunits of cohesin or condensin complexes respectively (Schubert et al., 2009, 2013). Given that both CRWN proteins and condensing/cohesion complexes affect chromocenter organization and chromosome compaction it might be interesting to further investigate whether a functional relationship exists between these complexes.

## Chromocenter Maintenance and Dynamics

Mutants impaired in factors involved in setting of epigenetic marks such as DNA methyltransferases (Soppe et al., 2002; Mathieu et al., 2007; Stroud et al., 2014) and histone K27 and K9 methyltransferases (Jacob and Feng, 2009; Yelagandula et al., 2014), in chromatin remodeling (Probst et al., 2003) or in chromatin assembly (Schönrock et al., 2006) affect heterochromatin organization in chromocenters. Furthermore, recent data suggest a role for the histone variant H2A.W in heterochromatin condensation into chromocenters based on its capacity to promote chromatin fiber-to-fiber interactions through its C-terminal end *in vitro* (Yelagandula et al., 2014) and accordingly *h2a.w* double or triple mutants show chromocenter decondensation. In addition to H2A.W, the linker histone H1 facilitates folding of the nucleosome into higher-order structures (Zhou et al., 2013 and references therein). The observation that some plant cells (such as the spore mother cells) show a drastic reduction in chromocenter compaction, concomitantly to H1 depletion (She et al., 2013; She and Baroux, 2015) suggests a role for the linker histone in pericentromeric chromatin organization, but a causal relationship remains to be established. During development, the organization of centromeric and pericentromeric sequences in chromocenters is dynamic (Benoit et al., 2013). For example during germination, chromocenter organization is lost 1 to 3 days after imbibition and only small and diffuse pre-chromocenters can be detected (van Zanten et al., 2011). Chromocenter assembly then takes place in cotyledons during a short time window between 3 and 5 days after germination (Mathieu et al., 2003; Douet et al., 2008; Bourbousse et al., 2015). Decondensation of chromocenters was also observed at later developmental stages such as during floral transition, when the plant undergoes reprogramming from vegetative to reproductive state, or during protoplast formation, which trigger a partial decondensation of 5S rDNA and 180 bp repeats (Tessadori et al., 2007a,b). Furthermore, the organization of chromocenters dynamically changes upon pathogen infection (Pavet et al., 2006), or under abiotic stresses (Probst and Mittelsten Scheid, 2015). As an example, chromocenters decondense during prolonged heat stress (Pecinka et al., 2010), which requires HEAT-INTOLERANT 4 (HIT4; Wang et al., 2013b). These dynamic changes in chromocenter organization during development or stress might reflect global chromatin changes, revealing the role of chromocenters in the organization of euchromatic loops in nuclear space thereby potentially contributing to gene expression regulation.

## A Role for Non-Coding RNA in Centromere Function?

Centromeric and pericentromeric regions are essential for chromosome segregation in mitosis and meiosis and help to structure chromosomes through the formation of chromocenters in interphase. These are complex functions requesting many factors including specific DNA sequences, deposition of histone variants and epigenetic marks, as well as chromatin organization in nuclear space. While centromeric and pericentromeric regions form a generally repressive chromatin environment, some of these repetitive elements are expressed at low level in specific tissues or developmental stages and processed by the RNAi pathway (May et al., 2005; Slotkin et al., 2009; Slotkin, 2010). In recent years, evidence for RNA in centromere regulation and function accumulated in different organisms (reviewed in Gent et al., 2012). Examples include a role for non-coding RNAs in heterochromatin assembly in fission yeast (Volpe et al., 2002), HP1 recruitment (Maison et al., 2002) and chromocenter organization (Probst et al., 2010) in mammals, as well as CenH3 deposition in mammals and Drosophila (Quénet and Dalal, 2014; Rošić et al., 2014). Furthermore, the passage of RNA polymerase II itself is critical for centromere function (Catania et al., 2015; Chen et al., 2015). Understanding the complex interplay between DNA sequence, transcription, non-coding RNA, chromatin and nuclear environment in centromere function in plants will be a major challenge for the future.

## Author Contributions

LS and MV contributed equally to the content and to the drafting of the manuscript. CT and AP edited the manuscript. All authors read and approved the manuscript.

### Conflict of Interest Statement

The authors declare that the research was conducted in the absence of any commercial or financial relationships that could be construed as a potential conflict of interest.
